# The Influence of Bubbles on the Perception Carbonation Bite

**DOI:** 10.1371/journal.pone.0071488

**Published:** 2013-08-21

**Authors:** Paul M. Wise, Madeline Wolf, Stephen R. Thom, Bruce Bryant

**Affiliations:** 1 Monell Chemical Senses Center, Philadelphia, Pennsylvania, United States of America; 2 Institute for Environmental Medicine, University of Pennsylvania, Philadelphia, Pennsylvania, United States of America; University of Montreal, Canada

## Abstract

Although many people naively assume that the bite of carbonation is due to tactile stimulation of the oral cavity by bubbles, it has become increasingly clear that carbonation bite comes mainly from formation of carbonic acid in the oral mucosa. In Experiment 1, we asked whether bubbles were in fact required to perceive carbonation bite. Subjects rated oral pungency from several concentrations of carbonated water both at normal atmospheric pressure (at which bubbles could form) and at 2.0 atmospheres pressure (at which bubbles did not form). Ratings of carbonation bite under the two pressure conditions were essentially identical, indicating that bubbles are not required for pungency. In Experiment 2, we created controlled streams of air bubbles around the tongue in mildly pungent CO_2_ solutions to determine how tactile stimulation from bubbles affects carbonation bite. Since innocuous sensations like light touch and cooling often suppress pain, we predicted that bubbles might reduce rated bite. Contrary to prediction, air bubbles flowing around the tongue significantly enhanced rated bite, without inducing perceived bite in blank (un-carbonated) solutions. Accordingly, though bubbles are clearly not required for carbonation bite, they may well modulate perceived bite. More generally, the results show that innocuous tactile stimulation can enhance chemogenic pain. Possible physiological mechanisms are discussed.

## Introduction

Although sweetness and alcohol are primary drivers of beverage choice, carbonation is also an important component of the majority of beverages consumed currently [1]. Recent work has offered some important insights into the unique and interesting sensation from carbonated beverages (e.g. [2]). However, the physiological basis of carbonation sensation has still not been fully elucidated.

The proximal stimulus for the pungent or painful aspect of carbonation is acidification of tissue and embedded trigeminal nerve endings due to enzymatic hydration of CO_2_ to carbonic acid by carbonic anhydrase (CA) 3–5. We know CA is required because inhibition of carbonic anhydrase specifically inhibits responses of peripheral trigeminal neurons [3], activation of c-fos in neurons of the trigeminal nucleus caudalis [6] and responses of wide dynamic range neurons in the trigeminal nucleus to CO_2_ [4]. Further, psychophysical studies demonstrate that inhibition of carbonic anhydrase specifically inhibits perceived carbonation bite [4], [6]. Carbonation bite is also attenuated by cross-desensitization with capsaicin, suggesting that sensation is mediated at least in part by polymodal nociceptors [7]. In particular, recent work in molecular biology strongly suggests that the transient receptor potential family member TRPA1 plays a major role in transduction of intraneuronal acidification in general, and intraneuronal acidification from CO_2_ in particular [2], [5].

Although the peripheral physiology of carbonation bite is becoming clearer, a belief that bubbles contribute to the nociceptive/irritating aspect of carbonation is still commonly expressed. The physics of bubble formation are discussed more fully elsewhere [8]. There has been speculation that bubbles contribute to the quality of carbonation sensation either as punctate sources of CO_2_ [9] or through nociceptive mechanical stimulation by the evolution or bursting of bubbles on the surface of the tongue [10], [11]. These possibilities remain untested. Although the physical force of bubbles may be too weak to directly stimulate mechanonociceptors embedded in the oral mucosa, light touch can modulate pain [12]–[15]. Thus, it seems plausible that innocuous mechanical stimulation from bubbles might modulate CO_2-_ induced nociception.

One might gain some insight by measuring carbonation sensation when bubbles are both present and absent. Simons and colleagues [4] referred to an unpublished study in which subjects rated carbonated beverages both at standard atmospheric pressure, at which bubbles could form, and at hyperbaric pressure, which purportedly suppressed formation of bubbles. Carbonated beverages were rated as pungent even at pressures that prevented formation of bubbles, suggesting that bubbles are not necessary for pungency. Unfortunately, since the study remains unpublished, detailed experimental procedures and results are not available. Thus, in Experiment 1, subjects rated carbonated water in a hyperbaric chamber both at normal atmospheric pressure and at hyperbaric pressure (2 atmospheres) to confirm that bubbles are not required for carbonation bite. Still, bubbles are clearly part of the normal sensory experience of carbonation. Thus, in Experiment 2, bubble formation was experimentally controlled by creating streams of air bubbles in mildly pungent CO_2_ solutions. Experiment 2 demonstrated that tactile stimulation modulates carbonation bite under more controlled conditions.

## Experiment 1

### Purpose

To determine whether bubbles forming in carbonated water are needed for carbonation bite, subjects evaluated carbonated water samples both at normal atmospheric pressure (at which bubbles can form) and at a higher atmospheric pressure (at which bubbles cannot form).

### Materials and Methods

#### Ethics statement

All research was conducted according to the principles expressed in the Declaration of Helsinki, and approved by an Institutional Review Board at the University of Pennsylvania. Subjects provided written, informed consent on forms approved by the Institutional Review Board.

#### Participants

Fourteen healthy adult nonsmokers (8 female, 6 male; mean age = 31.2 year, S.D. = 5.6) participated. All had experience in psychophysical experiments (e.g., rating the intensity of tastes or smells), and 6 had experience rating carbonation sensation in particular. Participants were recruited from among employees of the Monell Center and the surrounding community, and paid for their time. Absence of early stage pregnancy among participating women was verified using digital Digital Clearblue® Easy home pregnancy tests [16].

#### Stimulus materials

Stimuli were carbonated water samples, prepared from filtered water and presented at approximately 5 °C. Concentrations included 0.0 (blank), 1.5, 2.0, and 2.8 V_CO2_/V_water_ for the main study, with additional concentrations used for training purposes. For reference, 2.8 V/V is slightly lower than levels in typical carbonated soft drinks. The levels of dissolved CO_2_ and the solution temperature were chosen to cover a range of perceived intensities while allowing complete suppression of bubbles at 2.0 atm pressure in a hyperbaric chamber. Samples were prepared and bottled in 335 ml brown glass crown cap bottles.

#### Training

In the first of two training sessions, conducted in sensory testing facilities at Monell, participants received standard instructions in the use of the general Labeled Magnitude Scale, or gLMS [17]. The gLMS is a visual line scale with verbal descriptors including “no sensation,” “barely detectable,” “weak,” “moderate,” “strong,” “very strong,” and “strongest imaginable sensation of any kind.” The spacing of the descriptors was designed to provide ratio-level measurement [18]. Participants rated two aspects of carbonation sensation separately: “Bite” and “tickle/bubbles.” Bite was defined as the painful component of carbonation sensation (sting, burn, or pungency). Tickle/bubbles was defined as the non-painful component of carbonation sensation (bubbles on the tongue or in the mouth).

To help reduce inter-subject variability, subjects dipped the tips (∼ 1.5 cm) of their tongues into several exemplar stimuli. To exemplify cold with no carbonation bite, subjects held the tips of their tongues in 10°C, un-carbonated water. To exemplify carbonation bite with no bubbles, subjects held their tongues in 1.0 v/v carbonated water, which, after several seconds of exposure, gave rise to a mild but noticeable carbonation bite with no visible bubbles forming in the solution or around the tongue. To exemplify bubbles with no carbonation bite, subjects held the tip of the tongue in un-carbonated water over a porous liquid chromatography filter. Air was pumped through the filter to create air bubbles around the tongue (see Materials and Methods for Experiment 2, below). To demonstrate both bubbles and bite, subjects held their tongues in 3.2 v/v carbonated water, which gave rise to a sharp carbonation bite and noticeable bubbling. Finally, subjects rated both “Bite” and “Tickle/Bubbles” for both un-carbonated water and carbonated water at a range of concentrations. For each participant, experimenters verified that ratings were close to zero (“no sensation”) for blanks and increased with carbonation concentration. The first training session required about an hour.

The second training session, also conducted in sensory testing facilities at Monell, occurred between 2 and 5 days after the first session. Subjects were instructed not to eat or drink anything (except for water) for at least one hour before attending the session. Subjects were first re-introduced to the difference between bubbles and bite using 1.0 v/v carbonated water and air bubbles in un-carbonated water. Next, subjects rated all stimuli to be used in a hyperbaric chamber (see below), viz. 0.0, 1.5, 2.0, and 2.8 v/v, twice each in blocked random order. During each experimental trial, subjects placed the tips of their tongues in clear glass beakers containing 60 ml of liquid. After 10 s of holding the tongue tip as still as possible, subjects rated the intensity of both carbonation bite (the strongest sensation experienced during the 10 s) and tickle/bubbles. Next, after a pause of about 20 seconds, subjects were given fresh 40 ml samples of the same liquid. Subjects were instructed to drink the sample in two “gulps,” and focus on sensations felt in the back of the throat. Subjects then rated both Bite and Tickle/Bubbles in the throat. Subjects rated sensation by marking paper gLMS scales. At least 2 minutes elapsed between the end of one trial and the beginning of the next.

#### Hyperbaric chamber procedures

The third and final experimental session was conducted in a large, walk-in hyperbaric chamber at the Institute for Environmental Medicine at the University of Pennsylvania. The chamber comfortably accommodated up to 7 subjects, one Monell experimenter to administer the sensory test, and a chamber operator from the Institute for Environmental Medicine. Subjects completed two blocks of trials, each using procedures identical to those described for the second training session (above). In each block of trials, subjects rated 0.0, 1.5, 2.0, and 2.8 v/v carbonated water twice in blocked random order. One block of trials was conducted at normal (1 atm) pressure. The other block was conducted at 2 atm pressure (equivalent to a depth of 33 feet of sea water). Half the subjects (randomly assigned) rated samples at 1 atm first, whereas the other half rated samples at 2 atm first. In short, the design included two within-subjects variables (four levels of carbonation X two levels of atmospheric pressure) and one between-subjects variable (order of atmospheric pressure).

At the beginning of the higher pressure block, pressure was increased by between 1 and 2 psi per minute until a pressure of 2 atm was reached (after 5 to 7 min). This pressure was maintained for about 20 minutes, during which time sensory tests were conducted. After sensory testing, pressure was decreased slowly to normal levels. Some subjects experienced mild pain in the ears during pressurization, a common effect, but all were able to equalize pressure in their ears and reported being comfortable during the 20 min of sensory testing. Timing of the block of trials at normal pressure was the same. Subjects were given a break of about 30 minutes between blocks of trails.

#### Data analysis

One subject completed two training sessions but chose not to participate in the hyperbaric chamber session. A second subject was eliminated for failure to follow directions. Thus, the final sample for analysis included 12 subjects. Replicate ratings collected in the hyperbaric chamber were averaged within individuals using the arithmetic mean. Since distributions of intensity ratings tend to be positively skewed across subjects, all ratings were log-transformed before analysis (with the exception of ratings for the un-carbonated blanks, which were treated separately; see Preliminary analyses, below). Most effects of independent variables were assessed using analysis of variance (ANOVA) models. All analyses were conducted using Statistica software (Version 10, StatSoft), using an alpha value of <0.05.

### Results and Discussion

#### Preliminary analyses

In preliminary analyses, the between-subjects effects of gender and block order, i.e., subjects who made ratings at higher pressure first vs. those who made ratings at normal pressure first, failed to approach significance, as did all interactions involving these two variables. Accordingly, gender and block order were dropped from further analyses. Analyses of ratings for un-carbonated blanks were all essentially zero (closer to “no sensation” than “barely detectable”). In a series of one-sample t-tests of ratings of blanks vs. zero, p-values were all above 0.20. The fact that un-carbonated samples were rated as lacking bite and tickle/bubbles supports the validity of the ratings. To simplify analysis and interpretation, analyses below focus only on the carbonated samples.

#### Carbonation bite

**Figure 1:**
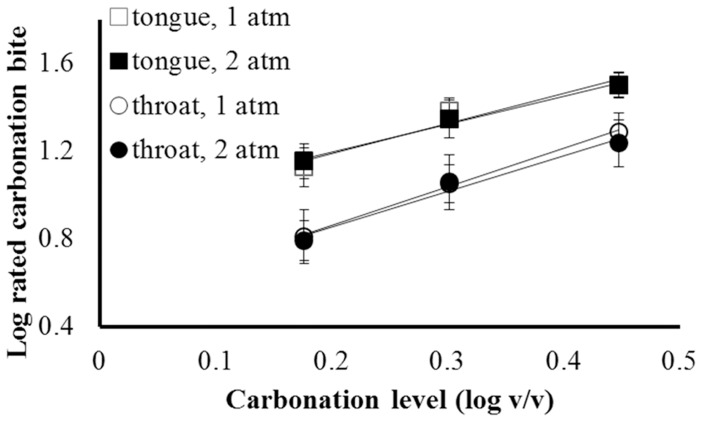
Log rated bite vs. log concentration, by anatomical site and atmospheric pressure. Error bars represent ±S.E.M. Lines are linear fits from least squares regression: 1) For tongue sensation at 1 atm, log(Intensity) = 1.37[log(Concentration)] +0.92, R^2^ = 0.94; 2) for tongue sensation at 2 atm, log(I) = 1.27[log(C)] +0.94, R^2^ = 0.99; 3) for throat sensation at 1 atm, log(I) = 1.76[log (C)] +0.51, R^2^ = 0.99; 4) For throat sensation at 2 atm, log(I) = 1.63[log(C)] +0.53, R^2^ = 0.97.

Rated bite was submitted to a 3-way, repeated measures ANOVA: Anatomical focus (tongue tip vs. throat) X Carbonation level (1.5, 2.0, and 2.8 v/v) X Pressure (ratings made at 1 atm vs. 2 atm). The main effect of Pressure and all interactions involving pressure failed to reach significance. Consistent with analyses, there was little or no difference between ratings made at 2 atm, at which bubbles could not (and, according to experimenter observations, did not) form, and ratings made at 1 atm, at which bubbles could form (Figure 1). Clearly, bubbles are not required for the perception of carbonation bite.

In contrast, the main effect of Carbonation level did reach significance, F (2, 22) = 38.91, p<0.00001, reflecting an expected concentration-response relationship (Figure 1). This clear association between the stimulus and reported sensation supports the validity of the ratings. The effect of Anatomical focus also reached significance, F (1, 11) = 44.53, p<0.0001. Ratings of sensation on the tongue tip were higher than for those of throat sensation. The interaction between Carbonation level and Anatomical focus was marginal, F(2,22) = 3.39, p = 0.052. Nominally, slopes of log bite vs. log concentration functions were flatter for tongue sensation (∼1.3) than for throat sensation (∼1.7). Both slopes fell within the range of published values for carbonation pungency [9]–[11], [19]–[21]. Differences between the tongue tip and throat could reflect differences in either the structure or innervation of the two tissues. For example, the transient receptor potential channel TRPA1, which seems to play an important role in transduction of pain from carbonation, is more densely expressed in the back of the throat in humans [2], [22]. Denser innervation by TRPA1-expressing fibers might be consistent with stronger rather than weaker sensation in the throat. However, the method of presentation could also play a role. Swallowed stimuli remained in contact with tissue only briefly relative to the steady presentation on the tongue tip, which could provide more time for temporal integration (Wise et al., 2003). Regardless, ratings at both anatomical locations show no effect of pressure, supporting the same conclusions regarding the contribution of bubbles to bite sensation (Figure 1).

#### Tickle/bubbles

**Figure 2:**
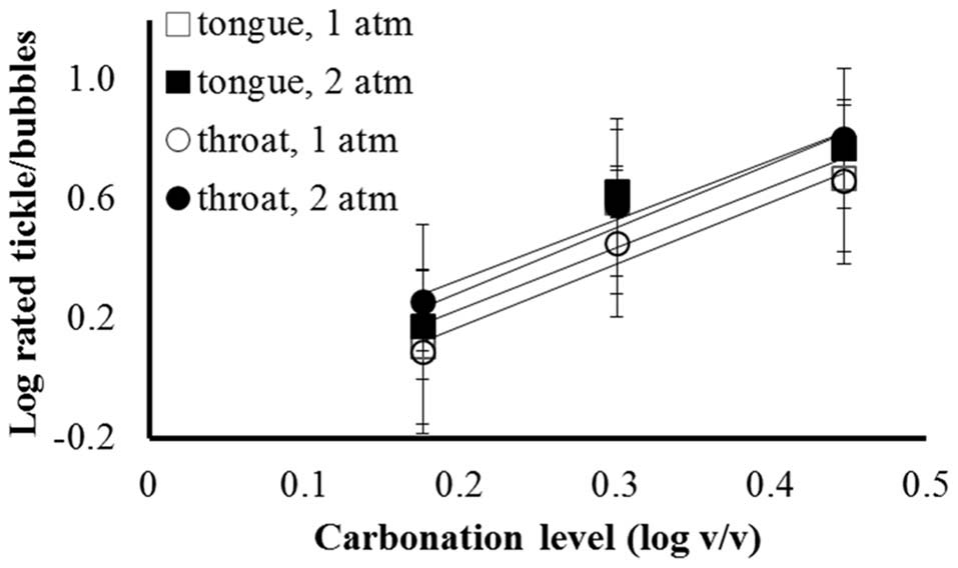
Log rated tickle/bubbles vs. log concentration, by anatomical site and atmospheric pressure. Error bars represent ±S.E.M. Lines are linear fits from least squares regression: 1) For tongue sensation at 1 atm, log(Intensity) = 2.04[log(Concentration)] - 0.18, R^2^ = 0.82; 2) for tongue sensation at 2 atm, log(I) = 2.16[log(C)] - 0.14, R^2^ = 0.89; 3) for throat sensation at 1 atm, log(I) = 2.08[log (C)] - 0.24, R^2^ = 0.96; 4) For throat sensation at 2 atm, log(I) = 2.00[log(C)] - 0.07, R^2^ = 0.98.

Ratings of tickle/bubbles were submitted to a 3-way ANOVA: Anatomical focus X Carbonation level X Pressure. As for rated bite, the main effect of pressure, and all interactions involving pressure, failed to reach significance. The only effect that did reach significance was Carbonation level, F (2, 22) = 11.73, p<0.001. Slopes of log intensity vs. log concentration functions were steeper (∼2.0) than were those for rated bite (Figure 2).

If ratings of tickle/bubbles were driven entirely by mechanical stimulation from carbonation bubbles, ratings on the tongue tip should have increased with carbonation level at 1 atm, but should have been close to zero at 2 atm (since no bubbles were observable). Expectations for rated throat sensation are less clear since we were unable to visualize solutions as they were taken into the mouth and swallowed. Still, the absence of an effect of Pressure, and the presence of a clear dose-response relationship for “tickle/bubbles” on the tongue tip show that ratings of tickle/bubbles were not entirely dependent on physical stimulation by CO_2_ bubbles.

Perhaps there are components of carbonation sensation (either painful or non-painful) triggered by tissue acidification that subjects mistakenly attribute to bubbles. For example, some human studies suggest that carbonated beverages can cause mild tingling or numbing [23]. Further, stimulation of taste cells by carbonic acid could give carbonated water a subtle taste [24]. Since bubbles are a salient aspect of carbonated beverages, people may attribute accompanying innocuous somatosensation or mild taste to bubbles. If so, these sensations may have fit subjects’ concept of bubbles, causing subjects to give positive ratings despite the training they received. Regardless, the finding that Pressure had little or no effect on any of the sensory ratings could imply that tactile stimulation from carbonation bubbles is not an important contributor to or modulator of carbonation bite. However, it is important to note that bubbles were not always apparent at 1 atm, either. In short, though this experiment clearly showed that bubbles are not required for carbonation bite, the experiment was not well suited to determine what, if any, subtle contributions bubbles might make since bubbles were not precisely controlled.

## Experiment 2

### Purpose

Experiment 1 showed that bubbles are not required to experience carbonation bite, but could not definitively determine whether bubbles modulate bite. Thus, we used air bubbles in solutions to simulate tactile stimulation from carbonation bubbles (we experimentally added bubbles while holding temperature and CO_2_ concentration constant). Since light touch often suppresses pain and itch [12], we hypothesized that flowing bubbles around the tongue would decrease rated carbonation bite.

### Materials and Methods

#### Ethics statement

All research was conducted according to the principles expressed in the Declaration of Helsinki, and approved by an Institutional Review Board at the University of Pennsylvania. Subjects provided written, informed consent on forms approved by the Institutional Review Board.

#### Participants

Fourteen healthy adult non-smokers (6 female, 8 male; mean age = 31.8 year, S.D. = 5.5) participated. All had experience in psychophysical experiments (e.g., rating the intensity of tastes or smells), and 8 had participated in Experiment 1.

#### Stimulus materials

Stimuli were carbonated water samples, prepared from filtered water and presented at approximately 10 °C. Concentrations included 0.0 (blank), 1.0, 1.6 V/V. The levels of dissolved CO_2_ and the solution temperature were chosen to provide noticeable carbonation bite under the sampling conditions (see Procedures, below), but to produce little or no bubbling (either in solution or around the tongue when dipped in solution). Samples were prepared and bottled (335 ml brown glass).

#### Apparatus

**Figure 3:**
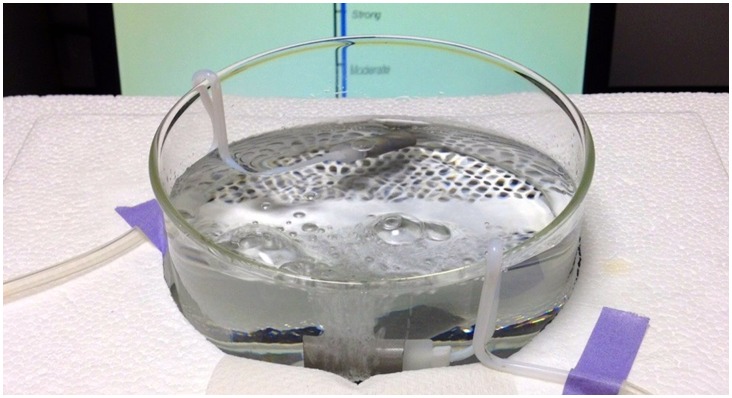
Beaker that contained the stimulus solutions. Inserted into the beaker were two HPLC filters through which air flowed to create bubbles. Subjects sampled the solutions by inserting the tip of their tongue over the near HPLC filter. Under the control of a solenoid valve, bubbles could be presented under the tongue (as shown, or away from the tongue (by directing air to the further HPLC filter). In the background, a computer monitor is visible (subjects could see it clearly). On the monitor is an LMS scale. Subjects continuously tracked bite intensity by moving a mouse that controlled the height of blue bar next to the LMS.

Carbonated water samples were presented in a shallow glass beaker (Figure 3). Two HPLC filters (2 µm pore size, Chromtech) were submerged in the liquid. Teflon® tubing connected the filters to an air source (compressed medical air). A 2-way Teflon® solenoid valve gated air flow, such that air passed through only one filter at a time. Flow rate was set at 240 ml/min, controlled by a rotameter. These conditions produced a column of bubbles in solutions that appeared (according to experimenter judgments) roughly comparable to the flow of bubbles in a freshly poured glass of seltzer water at room temperature.

Subjects dipped their tongue-tips (∼ 1.5 cm) into solution directly above one of the filters, near one edge of the beaker. The other filter was positioned on the opposite side of the beaker, away from the tongue. In this fashion, bubbles could be either introduced around the tongue, or away from the tongue. The second air-stone (away from the tongue) was included to control for any splashing that might impinge on the face. To control for noise (both from bubbling and the solenoid actuating) subjects wore noise-cancelling headphones during all judgments. Subjects continuously tracked the intensity of carbonation bite by moving a mouse, which in turn controlled the position of a cursor next to a virtual gLMS scale on a computer monitor. When subjects were in position with their attention fixed on the monitor, they were unable to see the beaker containing the stimulus-solution. Experimental timing and data collection were controlled by a custom program written using Labview® software (version 2011, National Instruments). The program recorded the position of the cursor (current rating of intensity) every second.

#### Training

Before formal collection of data, subjects completed two training sessions. In the first training session, subjects were given general instructions for the gLMS (regardless of whether they had used the scale before), and rated a solution of 1.6 V/V carbonated water that produced noticeable bite with little or no visible bubbling. They were instructed to regard the sensation occurring in this situation as “carbonation bite”. Subjects also experienced air bubbles applied to the tongue in un-carbonated water. Subjects were told that the water was un-carbonated, and that the bubbles were from air. Subjects rated the carbonated samples as having bite, but did not rate the un-carbonated water with air bubbles as having bite. During the second training session, subjects completed several practice trials (under the same conditions as the main experiment; see Procedures, below).

#### Procedures

**Figure 4:**
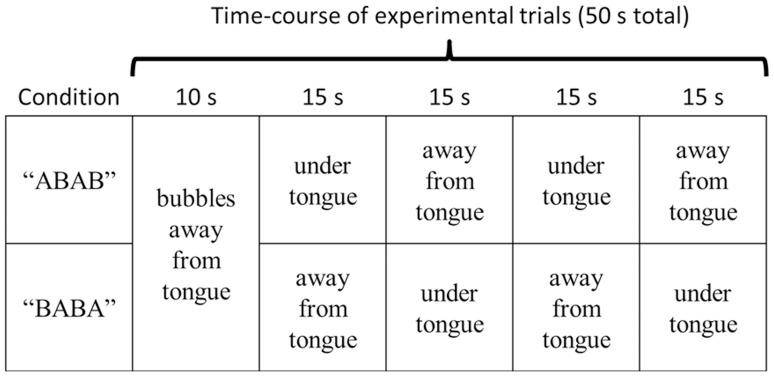
Illustration of order of bubble stimulation. Subjects continuously rated bite on the tongue tip. In both conditions, bubbles were away from the tongue (“off”) for the first 10 seconds. After this, the location of the bubbles (away (B) vs. under the tongue(A)) was changed after each 15 sec. The two orders of stimulation were counter balanced. for each subject.

During each experimental trial, subjects were instructed to immerse the anterior portion of their tongue and hold it as still as possible for 70 seconds. Subjects continuously tracked the intensity of carbonation bite during this time. The design included three Carbonation levels (0.0 V/V, 1.0 V/V, and 1.6 V/V) X 2 Bubble orders (ABAB vs. BABA; timing of bubbles during the trial, as explained below) X 2 replicate trials per condition (12 trials total). In both bubble orders, air was directed away from the tongue during the first 10 sec (“Off,” or no air bubbles around the tongue). After these initial 10 sec, bubble position alternated from under to away from the tongue every 15 seconds (Figure 4). In condition ABAB, air was directed to the filter under the tongue (position A) during the first 15 sec. period. In condition BABA, air was directed to the filter away (position B) from the tongue during the first 15 sec. period. The 12 trials were conducted in blocked random order over three experimental sessions. Four trials were collected per session, with breaks of at least 15 minutes between successive trials. Subjects were instructed not to eat or drink anything (except for water) for at least 1 hour before sessions.

#### Data analysis

Three subjects were excluded from analysis because they gave ratings of “moderate” or above to un-carbonated water, even at times when no air bubbles were added. Thus, the final sample for analysis included 11 subjects. Replicate ratings were averaged within individuals using the arithmetic mean. ANOVA models were used to evaluate effects of independent variables. Due to the substantial number of “0″ or “no sensation” ratings in the data set, log transformation was problematic. Data were therefore analyzed using untransformed values and ratings were averaged over 3 sec time-bins to smooth time-intensity curves and to limit errors caused by repeated statistical testing. All analyses were conducted using Statistica software (Version 10, StatSoft), with a criterion of p<0.05.

### Results and Discussion

Preliminary analyses found neither a significant main effect of subject gender nor significant interactions involving gender. Accordingly, data from men and women were pooled for further analysis. Ratings of carbonation bite were submitted to a 3-way, repeated measures ANOVA: Bubble order (ABAB vs. BABA) X Carbonation level (0.0, 1.0, and 1.6 V/V) X Elapsed time (24 time points, each a 3 sec average). The effect of Carbonation level was significant, F(1,10) = 33.62, p<0.000001. Ratings for the un-carbonated blank were essentially zero, with increasing ratings for higher levels of carbonation (ratings faithfully tracked physical concentration). The effect of Elapsed time also reached significance, F(23,230) = 7.28, p<0. 000001, reflecting a typical time-intensity curve (rise to peak followed by a slow decline in intensity). Further, the interaction between Elapsed Time and Concentration reached significance, F(46, 460) = 6.11, p<0.000001, indicating differences in the time-intensity curves across concentrations. All these effects are qualitatively similar to those for time-intensity ratings of pain from intra-nasal presentation of carbon dioxide [21].

**Figure 5:**
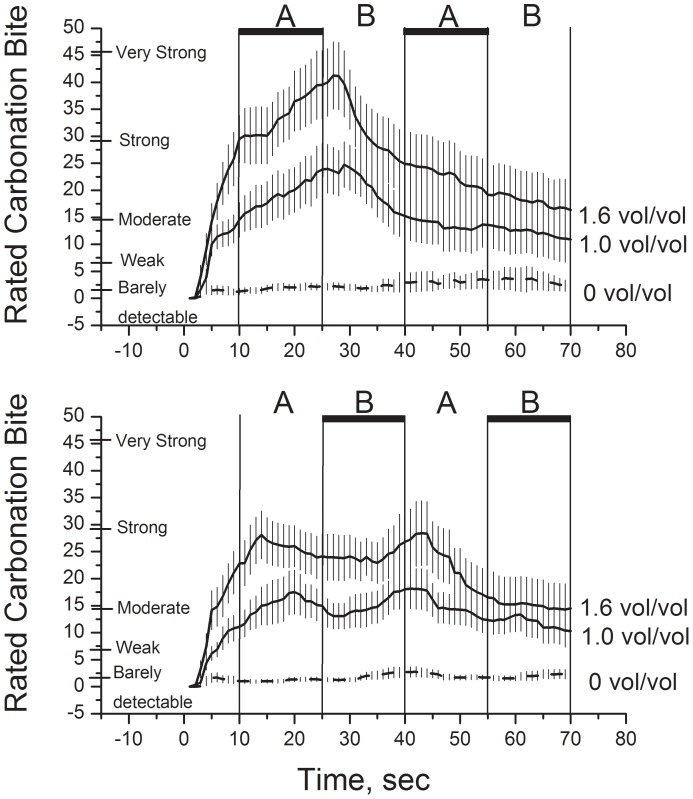
Rated carbonation bite over time (i.e., time-intensity curves) all conditions. The two bubble orders are represented in separate panels (top and bottom). Vertical lines represent transitions between time-periods in an experimental trial. Thick horizontal bars indicate periods in which air bubbles were flowing around the tongue. Error bars represent ±S.E.M.

The effect of Bubble order was significant, F(1, 10) = 6.74, p = 0.03. Ratings were slightly higher on average under the ABAB order. However, the main effect is perhaps less informative in the current design than the interactions involving Bubble order and Elapsed time, which would reflect perturbations of the time-intensity curves that are locked to presence of bubbles around the tongue. Accordingly, the significant interaction of Bubble order X Elapsed time, F(23, 230) = 4.06, p<0.000001, and the significant three-way interaction, F(46, 460) = 1.66, p<0.01, both indicate that bubbles flowed around the tongue modulated rated carbonation bite (Figure 5).

To further elucidate the effects of bubbles on rated bite, simple ANOVAs (Bubble order X Elapsed time) were performed separately on ratings for each level of carbonation (0.0, 1.0, and 1.6 V/V; Figure 5). Again, the interaction is of particular importance in analyses given the experimental design. For un-carbonated stimuli, neither the interaction nor the main effect of Bubble order reached significance (p>0.97 and p>0.39, respectively). There was a significant effect of Elapsed time, F(23,230) = 1.62, p = 0.04. However, the effect was weak and ratings never rose above ∼3 (just above “barely detectable”). Thus, flowing bubbles around the tongue had little or no effect on rated bite for the blank (un-carbonated) samples.

In contrast, for the 1.0 V/V samples, the interaction of Bubble order X Elapsed time was significant, F(23, 230) = 2.43, p<0.0001. Post hoc analysis of this interaction using Fisher’s LSD method showed significantly higher ratings of bite for the ABAB order at the 24, 27, 30 and 33 second time bins (all p<0.05). The main effect of time was also significant, F(23,230) = 4.91, p<0.000001, though the effect of Bubble order was not (p>0.38). The key two-way interaction also reached significance for the 1.6 V/V concentration, F(23, 230) = 2.94, p<0. 0001. Post hoc analysis of this interaction using Fisher’s LSD method showed significantly higher ratings of bite for the ABAB order at 12, 18, 21 24, 27, 30 and 33 second bins (all p<0.05). The main effects of Elapsed time, F(23,230) = 8.00, p<0.000001, and Bubble order, F(1, 10) = 4.97, p<0.05, also reached significance. Thus, bubbles flowing around the tongue modulated perception of bite for the two carbonated samples, but had little or no impact on ratings of un-carbonated samples.

**Figure 6:**
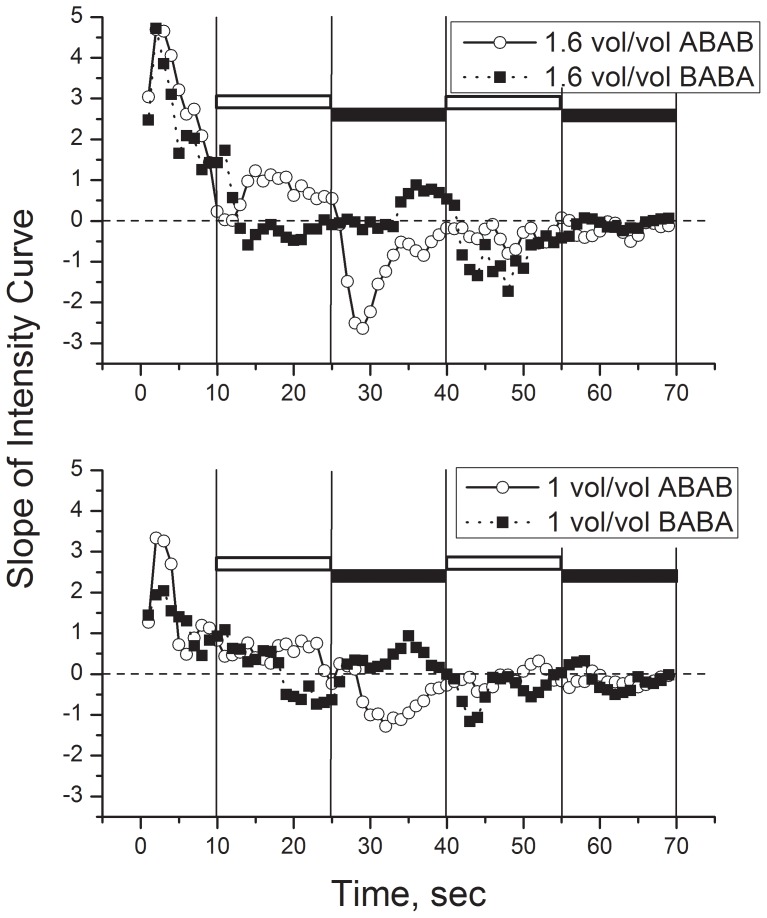
Slopes (three-point moving average) of time-intensity curves over time, indicating rate of change of bite sensation. Top panel: data for the higher level of carbonation (1.6 v/v). Bottom panel: data for the lower level of carbonation (1.0 v/v). In each panel, the open horizontal bars indicated time periods during which bubbles flowed around the tongue for the ABAB condition, and filled horizontal bars indicate time periods during which bubbles flowed around the tongue for the BABA condition. Ratings of bite in ABAB order trials are indicated by open circles while ratings of bite during BABA order trials are indicated by filled squares.

The significant interaction of Bubble Order over time is illustrated in Figure 5. For both Orders, ratings of bite increased during the first 10 sec (bubbles away from the tongue) only for carbonated stimuli. Between 11 to 25 seconds, ratings peaked and began a decrease for order BABA (Figure 5 bottom; no bubbles during this period), following a typical time-intensity curve [21]. However, for order ABAB (Figure 5 top; bubbles switched to under the tongue from 11 to 25 s), ratings continued to increase. Thus, the bubbles presented under the ABAB condition during this period appeared to enhance rated bite. From 26 to 40 seconds, rated intensity dropped sharply for the ABAB condition a few seconds after the bubbles were moved away from the tongue (Figure 5 top). Similarly, for the BABA condition, rated intensity began to increase after the bubbles were moved to under the tongue (Figure 5 bottom). Again, the result is consistent with bubbles enhancing rated carbonated bite. For both Bubble order conditions, the effects of the second period of bubbles under the tongue (41–55 s for ABAB, 56–70 s for BABA) were less obvious. Another way to view the data is the rate of change in intensity over time. Figure 6 (top for 1.6 v/v, bottom for 1.0 v/v) illustrates the slope of the curves in Figure 5 (3-point moving average of the slope). Inspection of the slopes suggests that, in the absence of bubbles applied to the tongue, rated bite peaks between 10 and 20 seconds after dipping the tongue (filled squares Fig. 6, top and bottom, curves passes through zero) for both concentrations of CO_2_. With bubbling present the decline does not occur until after cessation of bubbles (open circles, Fig. 6 top and bottom, 25–30 seconds). In trials in which bubbling was delivered between 25 and 40 sec (BABA), the slope reversed from negative to positive between 25 and 35 sec (closed squares) indicating an increase in rated Bite. The rated intensity peaked and declined immediately following cessation of bubbling. The effects of the second period of bubbling on rated Bite intensity are less clear. There is an indication of a possible increase in bite induced by bubbling in the 1.0 v/v condition.

For both the 1.0 and 1.6 V/V stimuli, changes in rated bite lagged the first introduction of bubbles. Of course, subjects would require some time to notice and evaluate changes in sensation and to execute the motor response of moving the mouse. However, delays of up to about ∼10 sec before clear perturbations in the time-intensity curves (e.g., 1.6 V/V, BABA curve in Figure 5) are longer than one would expect considering the limitations imposed by response-time alone. We have no definitive explanation for these delays (but see “What is the source of enhancement by air bubbles?” under the General Discussion for further discussion).

Regarding the three subjects excluded from analyses because they attributed considerable carbonation bite to un-carbonated samples (despite training), it is unclear what sensation these subjects rated. Visual stimulation might have played a role. Subjects could not easily see the stimulus solutions while their attention was on the virtual rating scale, but subjects could see bubbles as they took position to prepare for a trial. Turbulence from air bubbles might also have caused water droplets to hit subjects’ faces. Such sensations could have influenced some subjects to give positive ratings.

## General Discussion

Though acidification of tissue and nerve endings is a key proximal stimulus for carbonation bite, bubbles are often thought to be a vital component of carbonation sensation. Indeed, the fact that many people attributed bubbles to carbonated solutions in the absence of bubbles (Experiment 1) may show how closely linked bubbles are to the concept of carbonation. However, the main results of Experiment 1 showed that subjects rated carbonation bite equally strong in CO_2_ solutions with (low pressure) and without (higher pressure) the presence of bubbles. The results provide a definitive demonstration that bubbles are not required for carbonation bite. Indeed, the fact that ratings were equal under the two pressure conditions might imply that bubbles make no contribution at all. However, Experiment 1 was not designed to directly test the possibility of bubbles modulating nociception.

In Experiment 2, we presented controlled streams of air bubbles around the tongue. We hypothesized that the tactile stimulation associated with bubbles in carbonated beverages would decrease carbonation bite in the same manner that innocuous touch [25] or mild cooling [26] inhibit itch, mild irritation, or pain. However, the opposite was observed: perceived carbonation bite increased after the onset of bubbling. The effect appeared to be stronger at higher carbonation levels, and was robust during the first application of bubbles (though appeared weaker or absent on subsequent applications of bubbles). In un-carbonated water, bubbles did not induce appreciable levels of carbonation bite. That bubbles did not impart bite to un-carbonated water argues against a simple scaling bias, i.e., that subjects simply included bubble sensation in ratings of bite [27], [28]. Rather, the results suggest some important interaction between carbonation bite and tactile stimulation.

### What is the source of enhancement by air bubbles?

Interactions between carbonation and bubbles may have a physico-chemical component. For example, bubbles may effectively stir the liquid layer near the surface of the tongue, maintaining a higher concentration gradient of CO_2_ at the surface of the tissue than in an unstirred condition, and thereby facilitating diffusion of CO_2_ into the oral mucosa. In unstirred conditions, there would normally be a layer of solution at the surface of the mucosa that is slightly depleted of CO_2_ due to diffusion into the mucosa; stirring could maintain the maximal concentration gradient at the mucosa by replacing the depleted layer with fresh solution from the bulk phase. This possibility is not easy to test experimentally in humans, though studies could be done on tongue tissue using pH sensitive dyes in conjunction with confocal microscopy to visualize the dynamics of proton concentration in tissue at the level of trigeminal nerve endings. Dynamics of tissue pH might also explain some of the lag between switching bubbles to under the tongue and subsequent perturbations of the time-intensity curves in Experiment 2, though it seems unlikely that pH dynamics could explain all of the 5–10 sec delays we observed. The reader should also note that our model bubbles were created using medical air rather than CO_2_, which could alter the biophysics of diffusion relative to actual carbonation bubbles. Thus, our simple model system, designed to approximate tactile sensations from carbonation bubbles, may not be a good model of carbonation bubbles in other ways.

Physicochemical explanations cannot explain the finding that the enhancing effect of bubbles was greatly diminished during the second application of bubbles. This decrement in enhancement could be due to either the effects of the prior tactile stimulus or continued exposure to CO_2_. If the mechanism of enhancement is desensitized, one might expect enhancement of bite if the second presentation of bubbles were either longer or more intense. Regardless, since subjects continue to perceive carbonation bite throughout the 70 sec trials, we speculate that the mechanism mediating enhancement by bubbles, whether peripheral or central, is separate from the primary transduction mechanisms for carbonation bite.

Regarding possible physiological mechanisms, the observed enhancing effect of bubbles on carbonation bite might be related to allodynia, a condition wherein innocuous stimuli cause pain. In some cases, allodynia is due to sensitization of peripheral nociceptors by inflammation [29]. Accordingly, tissue acidification from CO_2_ may sensitize nociceptors to innocuous tactile stimulation by bubbles, analogous to inflammatory sensitization of TRPA1-expressing afferents to mechanical stimulation [30].

In other cases, allodynia may be due to more central effects, such as inhibition of circuits that normally suppress nociceptive input at the level of the spinal cord (e.g. [31]). However, many interactions between innocuous tactile stimuli and existing pain are inhibitory [32]. Of course, CO_2_ may be a special case, exciting a unique set of neural pathways. If so, then tactile enhancement of bite should be specific to CO_2_ (and similar stimuli), and not generalize to other irritants like capsaicin or ethanol. Several experiments come immediately to mind. Perhaps the closest stimuli to CO_2_ would be organic acids capable of diffusing into the oral tissue, such as short chain fatty acids. The mild bite induced by butyric or pentanoic acid on the tongue should also be enhanced by bubbles if enhancement is a property of acid-sensitive, nociceptive pathways. Further, if the enhancing effect of bubbles is particular to pathways that express TRPA1 [2], then the bite of cinnamaldehyde, allyl isothiocyanate, and other agonists of TRPA1 should also be enhanced by bubbles.

More generally, very little is known regarding interactions between chemesthesis and mechanosensation. The current study provides new information using carbonated water, a simple but behaviorally relevant model system. Future studies can continue to examine how chemical and mechanical stimuli interact to shape somatosensory perception, with the working hypothesis that innocuous tactile stimulation enhances chemogenic pain. Carbonated beverages might also provide good model systems with which to explore how other modalities modulate somatosensory components of flavor. Actual beverages include taste, aroma, color, motion (streaming bubbles) and sound (fizzing), all of which contribute to the perception (and concept) of carbonated beverages. Therefore, as is becoming increasingly clear from studies of flavor, we must understand how all sense modalities interact to gain a full understanding of how we experience common objects like beverages [33]–[35].
